# Adjuvant Chemoradiotherapy Versus Adjuvant Chemotherapy for Stage III Gastric or Gastroesophageal Junction Cancer After D2/R0 Resection

**DOI:** 10.3389/fonc.2022.916937

**Published:** 2022-07-12

**Authors:** Jinming Shi, Wenzhe Kang, Yuan Tang, Ning Li, Liming Jiang, Lin Yang, Shulian Wang, Yongwen Song, Yueping Liu, Hui Fang, Ningning Lu, Shunan Qi, Bo Chen, Yexiong Li, Yantao Tian, Jing Jin

**Affiliations:** ^1^ Department of Radiation Oncology, National Cancer Center/National Clinical Research Center for Cancer/Cancer Hospital, Chinese Academy of Medical Sciences and Peking Union Medical College, Beijing, China; ^2^ Department of Pancreatic and Gastric Surgery , National Cancer Center/National Clinical Research Center for Cancer/Cancer Hospital, Chinese Academy of Medical Sciences and Peking Union Medical College, Beijing, China; ^3^ State Key Laboratory of Molecular Oncology and Department of Radiology, National Cancer Center/National Clinical Research Center for Cancer/Cancer Hospital, Chinese Academy of Medical Sciences (CAMS) and Peking Union Medical College (PUMC), Beijing, China; ^4^ State Key Laboratory of Molecular Oncology and Department of Medical Oncology, National Cancer Center/National Clinical Research Center for Cancer/Cancer Hospital, Chinese Academy of Medical Sciences (CAMS) and Peking Union Medical College (PUMC), Beijing, China; ^5^ Department of Radiation Oncology, National Cancer Center/National Clinical Research Center for Cancer/Cancer Hospital & Shenzhen Hospital, Chinese Academy of Medical Sciences and Peking Union Medical College, Shenzhen, China

**Keywords:** adjuvant chemoradiotherapy, adjuvant chemotherapy, gastroesophageal junction cancer, gastric cancer, survival analysis

## Abstract

**Purpose:**

To compare the survival benefit in the adjuvant chemoradiotherapy (CRT) group and chemotherapy (CT) group for stage III gastric or gastroesophageal junction (GEJ) cancer after D2/R0 resection.

**Methods and Materials:**

From January 2011 to May 2018, 819 patients (CRT group: 215 patients, CT group: 604 patients) diagnosed as pathological stage III after D2/R0 resection were retrospectively collected and the survival and recurrence patterns were analyzed. The baseline characteristics were balanced based on propensity score matching (PSM). The survival benefit was compared between two groups using Kaplan–Meier analysis and Cox regression model.

**Results:**

The 5-year overall survival (OS) rate in the CRT group was significantly higher than that in the CT group whether before or after the PSM. The multivariate Cox regression analysis identified the significant poor OS in patients with advanced TNM stage (P < 0.001) and patients who did not receive the adjuvant CRT (P = 0.008). For the recurrence patterns, 85 (39.5%) patients in the CRT group and 300 (49.7%) patients in the CT group were diagnosed as recurrence (P = 0.011). The regional recurrence in the CRT group was less than that in the CT group (20.5% vs. 35.1%, P = 0.028).

**Conclusion:**

For patients diagnosed as stage III gastric cancer or gastroesophageal junction cancer, the addition of adjuvant chemoradiotherapy will significantly improve the overall survival and regional control.

## Introduction

Gastric cancer is the fifth common cancer and ranks fourth in mortality worldwide (1). In China, about 70% of newly diagnosed gastric cancer patients are stage III whose prognoses are poor (2). For stage III gastric cancer or gastroesophageal junction (GEJ) cancer, a multidisciplinary approach is important (3). In the INT0116 study, adjuvant chemoradiotherapy (CRT) significantly improved the overall survival (OS) rate compared with patients who had surgery alone or gastric cancer patients who received D0–D1 resection (4). With the rapid development of surgery and chemotherapy (CT), D2 resection has become dominate and adjuvant chemotherapy has improved the survival time than surgery alone.

In the CRITICS study (5), the addition of adjuvant CRT failed to improve the OS rate compared with perioperative chemotherapy. We found that the completion rate of adjuvant treatment was less than 60%. In the ARTIST study (6, 7), adjuvant CRT reduced the local recurrence rate than the chemotherapy group for locally advanced GC cancer after D2 resection, but there was no significant difference in the OS rate between the two groups. Subgroup analysis showed that node-positive and intestinal-type GC patients can benefit from adjuvant CRT. However, the proportion of stage I–II patients in the ARTIST study was 60%, which did not conform to the distribution of gastric cancer in China. In the ARTIST II study (8), the adjuvant CRT group failed to improve disease-free survival compared with the CT group for lymph node-positive GC patients who received D2 resection. Moreover, subgroup analysis was not reported and follow-up time was limited. In some retrospective clinical trials, adjuvant CRT can further benefit for high cancer burden patients (9). Therefore, the value of adjuvant CRT is still controversial.

For further exploring the value of adjuvant CRT, we design this retrospectively clinical trial to evaluate if adjuvant CRT can benefit GC or GEJ cancer patients with stage III after D2/R0 resection.

## Methods and Materials

### Eligibility Criteria

Patients who met the following criteria were enrolled in this study: (1) age 18 to 80 years; (2) received D2/R0 gastrectomy from January 2011 to May 2018; (3) histologically diagnosed as Siewert II or III gastroesophageal junction (GEJ) or middle to distal gastric cancer; (4) pathologically diagnosed as stage III according to the 8th edition of the AJCC staging system. The exclusion criteria were as follows: (1) patients who received neoadjuvant chemoradiotherapy or chemotherapy; (2) patients who received R1 or R2 resection; (3) the follow-up time was less than 30 days after surgery; (4) patients diagnosed as gastric stump carcinoma when receiving surgery. According to adjuvant therapy, patients were divided into adjuvant CRT group and adjuvant CT group.

### Treatment

(1) Adjuvant chemotherapy group: patients received 4–6 cycles of adjuvant chemotherapy within 6–8 weeks after D2 gastrectomy. The chemotherapy regimens contained the following: (1) oxaliplatin and S-1 (SOX); (2) oxaliplatin and capecitabine (CapeOX); (3) cisplatin and 5-fluorouracil (5-FU); (4) paclitaxel and tegafur; (5) single-drug regimen such as tegafur, docetaxel, and capecitabine.

(2) Adjuvant chemoradiotherapy group: patients received 4–6 cycles of adjuvant chemotherapy as chemotherapy group firstly and then received CRT. Before the CT simulation, patients were required to fast for 4 to 6 h and drink 300 ml water which contained a contrast agent to visualize the small intestine. During the enhanced CT simulation, patients were required to lay on the bed with their arms crossed above the heads. The scanning was from the clavicle to the fifth lumbar vertebra. Patients received 45 to 50.4 Gy with 25 to 28 fractions using either intensity-modulated radiation therapy (IMRT) or volumetric modulated arc therapy (VMAT) with a 6-MV photon beam. The definition of target volume mainly referred to the EORTC guideline (10). The clinical target volume (CTV) included tumor bed, anastomosis site, and regional lymph nodes issued by the JCGA guideline (11). Considering the tumor motion and setup error, the planning target volume (PTV) was 0.5 to 1.0 cm expanded from the CTV. Ninety-five percent of the PTV should be given the prescribed dose. Concurrent chemotherapy contains the continuous intravenous infusion of 5-FU or orally capecitabine or S-1.

### Follow-Up

After completing the treatment, patients were followed up every 3 months in the first 2 years, and every 6 months for 3 to 5 years and yearly thereafter. Physical examination, blood routine, biochemical test, tumor biomarkers, abdomen and pelvis computed tomography scans, and gastroscopy were required during the follow-up. Investigators collected information of recurrence and survival by telephone or hospital follow-up information.

### Definition of Recurrence

Tumor recurrence was affirmed by biopsy or imaging. Based on the first place of recurrence during the follow-up, recurrences were categorized as local recurrence, regional recurrence, and distant recurrence in this study. Local recurrence was defined as recurrence in the anastomosis, tumor bed, or remnant stomach. Regional recurrence was defined as recurrence in the regional lymphatic drainage (11, 12) within the radiotherapy target field in the CRT group or the assumed radiotherapy target field in the CT group. Distant recurrence was defined as metastases in the peritoneum, pleura, solid organ, distant lymph nodes, or abdominal wall metastasis.

### Statistical Analysis

All analyses were conducted using SPSS Statistics 22.0 (Chicago, IL, USA). To reduce the selection bias between the CRT group and CT group, one-to-one propensity score matching (PSM) was performed. The Pearson’s chi-square test or Fisher’s exact test was used to analyze the differences in baseline characteristics between two groups. The Kaplan–Meier method and Cox regression model were used to analyze the survival benefit. The recurrence-free survival time was defined as the time from the date of surgery to the first occurrence of recurrence or death. The overall survival time was defined as the time from surgery to death. *P*-value <0.05 was considered as statistically significant.

## Result

### Patient Characteristics

A total of 819 patients with stage III gastric cancer or GEJ cancer were enrolled retrospectively. There were 215 patients and 604 patients in the CRT group and CT group, respectively. Among them, nearly 95% of patients were stage pT3–4 and nearly 80% of patients were stage pN3. Compared with the CRT group, patients in the CT group were older before the PSM (*P* < 0.001). After the PSM, the age was balanced between two groups (P = 0.149). In the CRT group, the primary site of the tumor was closer to the mid-distal stomach compared with that in the CT group whether before or after the PSM. The patients’ characteristics between two groups are listed in **Table 1**.

**Table 1:** Baseline characteristics of patients in the CRT group and CT group.

	CRT group (N = 215)	CT group			
		Before PSM (N = 604)	P-value	After PSM (N = 215)	P-value
**Age**					
Median (range)	53 (22–82)	59 (23–82)	<0.001	56 (23–80)	0.149
**Gender**					
Male	153 (71.2)	434 (71.9)	0.847	162	0.327
Female	62 (28.8)	170 (28.1)		53	
**Primary site**					
Upper	55 (25.6)	229 (37.9)	<0.001	69 (32.1)	0.006
Body	28 (13.1)	85 (14.1)		30 (14.0)	
Pylorus	111 (51.5)	192 (31.8)		77 (35.8)	
≥2/3 of stomach	21 (9.8)	98 (16.2)		39 (18.1)	
**No. of LNs examined**					
Median (range)	32 (9–84)	33 (10–181)	0.736	33 (12–94)	0.686
**No. of positive LNs**					
Median (range)	11 (1–48)	10 (0–71)	0.314	10 (0–43)	0.241
**Grade**					
Well differentiated	1 (0.5)	6 (1.0)	0.080	0	0.144
Moderately differentiated	16 (7.4)	70 (11.6)		26 (12.1)	
Moderately to poorly differentiated	38 (17.7)	138 (22.8)		50 (23.3)	
Poorly differentiated	160 (74.4)	390 (64.6)		139 (64.6)	
**T-stage**					
T1–2	7 (3.3)	27 (4.5)	0.581	13 (6.0)	0.400
T3	82 (38.1)	237 (39.2)		87 (40.5)	
T4a	119 (55.3)	307 (50.8)		104 (48.4)	
T4b	7 (3.3)	33 (5.5)		11 (5.1)	
**N-stage**					
N0–2	45 (20.9)	140 (23.2)	0.668	44 (20.5)	0.868
N3a	111 (51.7)	287 (47.5)		108 (50.2)	
N3b	59 (27.4)	177 (29.3)		63 (29.3)	
**TNM stage**					
IIIA	45 (20.9)	154 (25.5)	0.120	52 (24.2)	0.802
IIIB	112 (52.1)	266 (44.0)		99 (46.0)	
IIIC	58 (27.0)	184 (30.5)		64 (29.8)	

### Survival Analysis

**Figure 1:**
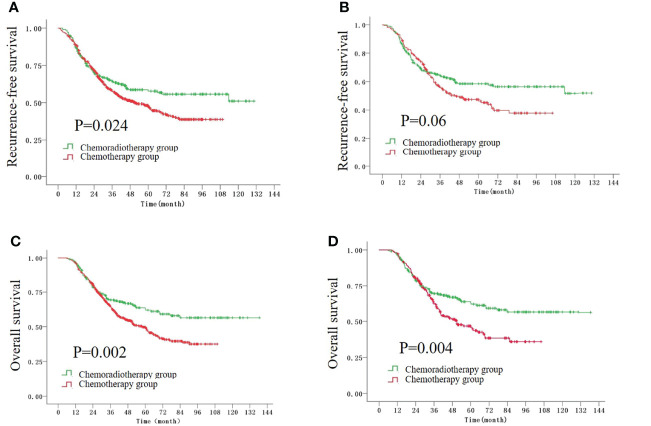
The Kaplan–Meier survival curves for RFS time before **(A)** and after **(B)** the PSM, and the curves for OS time before **(C)** and after **(D)** the PSM.

Before the PSM, the 5-year recurrence-free survival (RFS) rates were 57.7% and 47% in the CRT group and CT group, respectively (P = 0.024, **Figure 1A**). The 5-year overall survival (OS) rate in the CRT group was significantly higher than that in the CT group (62.8% vs. 49.4%, P = 0.002, **Figure 1C**). After the PSM, the 5-year OS rate was still higher in the CRT group compared with that in the CT group (62.8% vs. 45.7%, P = 0.004, **Figure 1D**), while there was a trend to improve the 5-year RFS rate in the CRT group (57.7% vs. 46.3%, P = 0.06, **Figure 1B**).

### Recurrence Patterns

**Figure 2:**
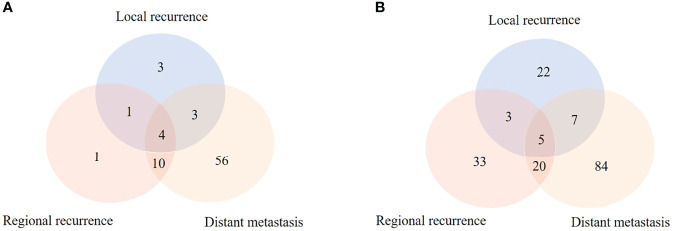
The recurrence patterns in the CRT group **(A)** and CT group **(B)**.

During the follow-up by telephone or specific recurrence information, 85 (39.5%) patients in the CRT group and 300 (49.7%) patients in the CT group were diagnosed as recurrence (P = 0.011). However, among these patients, 7 (8.2%) patients in the CRT group and 126 (42.0%) patients in the CT group were unable to obtain specific recurrence information due to the follow-up by telephone. For the local recurrence composition ratio, no significant difference was found in the CRT group and CT group (14.1% vs. 20.1%, P = 0.213). The regional recurrence composition ratio in the CRT group was less than that in the CT group (20.5% vs. 35.1%, P = 0.028), while the distant recurrence composition ratio was higher in the CRT group compared with the CT group (93.6% vs. 66.7%, P = 0.007) (**Figure 2**). From **Table 2**, we found that peritoneal metastasis (39.7%) was more common in the CRT group, while regional recurrence (35.1%) was the main failure pattern followed by solid organ metastasis (29.3%) in the CT group.

**Table 2:** The distribution of recurrence in the CRT group and CT group.

Recurrence site	CRT group		CT group	
	No. of patients	% of recurrence patients (n = 78)	No. of patients	% of recurrence patients (n = 174)
Local recurrence				
Remnant stomach	6	7.7%	6	3.4%
Anastomosis site	5	6.4%	29	16.7%
Regional recurrence	16	20.5%	61	35.1%
Distant metastasis				
One site				
Peritoneum	31	39.7%	30	17.2%
Pleura	4	5.1%	5	2.9%
Solid organ	19	24.4%	51	29.3%
Distant LNs	4	5.1%	14	8.0%
Abdominal wall metastasis	3	3.8%	4	2.3%
≥Two sites				
Peritoneum + solid organ	5	6.4%	3	1.7%
Solid organs	5	6.4%	8	4.6%
Peritoneum + distant LNs	2	2.6%	1	0.6%

### Univariate and Multivariate Analysis of Overall Survival

From **Table 3**, five variables which included age, T stage, N stage, TNM stage, and whether they received CRT were shown related to the overall survival in the univariate analysis. In the multivariate analysis, five variables were included in the Cox regression. The multivariate analysis showed that advanced TNM stage (P < 0.001) and not being able to receive the chemoradiotherapy treatment were the significant risk factors for OS.

**Table 3:** Univariate and multivariate Cox regression analyses for overall survival.

Variable	Univariate analysis		Multivariate analysis	
	HR (95% CI)	P-value	HR (95% CI)	P-value
Gender				
Male	1	0.548		
Female	0.931 (0.737–1.176)			
Age				
<60	1	**0.018**	1	0.089
≥60	1.281 (1.044–1.573)		1.200 (0.972–1.481)	
Location				
GEJ	1	0.454		
Non-GEJ	0.923 (0.747–1.139)			
Grade				
Poorly differentiated	1	0.661		
Non-poorly differentiated	0.933 (0.685–1.272)			
T stage				
T1–2	1	**0.015**	1	0.257
T3–4	2.391 (1.187–4.818)		1.654 (0.693–3.947)	
N stage				
N0–2	1	**<0.001**	1	0.977
N3	2.075 (1.551–2.775)		0.986 (0.366–2.656)	
TNM stage				
IIIA	1	**<0.001**	1	**<0.001**
IIIB	1.907 (1.407–2.584)		1.844 (0.692–4.915)	
IIIC	2.915 (2.135–3.982)		2.769 (1.013–7.570)	
CRT				
Treated	1	**0.003**	1	**0.008**
Untreated	1.474 (1.146–1.897)		1.424 (1.096–1.849)	

The bold P-value less than 0.05 was considered as statistically significant.

## Discussion

As D2 resection becomes a standard surgery method for locally advanced GC or GEJ cancer, the value of adjuvant CRT has been questioned. In this retrospective clinical study, we have demonstrated that the addition of CRT to the adjuvant CT can improve the overall survival rate significantly for patients with stage III after D2 resection. Our result suggests a subpopulation of GC patients which may benefit from adjuvant CRT.

As the landmark of adjuvant CRT for gastric cancer, INT0116 firstly demonstrated that the addition of adjuvant radiotherapy can improve the OS compared with surgery alone after 10 years of follow-up (4), while in the INT0116 study, more than 90% of patients received D0 or D1 resection. With the development of surgery technology, D2 resection is related with a better local control and survival benefit than D1 resection and has been recommended for GC cancer especially in Asia (13). In the ARTIST study, adjuvant CRT and adjuvant CT were compared after D2 resection and 230 patients and 228 patients were enrolled respectively. After 7 years of follow-up, no significant difference was seen in the OS (P = 0.484) and DFS (P = 0.0862) between the CRT group and CT group (6). However, in the subgroup analysis, lymph node positive (P = 0.04) and intestinal subtype by Lauren classification (P = 0.01) were proved to improve the 3-year DFS in the CRT group compared with the CT group (7), while in the ARTIST study, 60% of patients were stage IB to II, which was not consistent with the distribution of tumor stages in China. Recently, the result of ARTIST II has proved that the adjuvant CRT group failed to improve the survival compared with the CT group for lymph node-positive patients after D2 resection (8), while the recruitment of fewer patients than planned reduced the statistical power and the median DFS was not reached. What is more, more than 30% of patients were stage II. We consider that adjuvant CRT may benefit patients with high cancer burden.

To further explore the subpopulation of GC patients who might benefit from CRT, Zhou et al. found that adjuvant CRT can improve the DFS for stage N3 GC patients (14). Other retrospective clinical studies also demonstrated that adjuvant CRT can benefit the high lymph node burden patients (15, 16). For the stage III patients, Ma et al. (17) enrolled 415 GC patients (CRT group: 135 patients, CT group: 280 patients) after D2 resection and found the significant OS benefit in the CRT group. What is more, Peng et al. (9) enrolled 337 patients with stage IIIc who received CRT (124 patients) or CT (213 patients) and found that the addition of adjuvant chemoradiotherapy was associated with a significant benefit in both OS and DFS. Similar to the previous studies, in our study, we enrolled 819 patients (CRT: 215 patients, CT: 604 patients) and found that the addition of adjuvant CRT can improve the OS rate for stage III GC patients in the Kaplan–Meier survival analysis and the result is still stable whether in the PSM analysis or in the multivariate analysis. The RFS rate was significantly higher in the CRT group compared with the CT group before the PSM, while after the PSM, we consider that adjuvant CRT has the possibility to improve the RFS.

For the exploration of recurrence patterns after radiotherapy, 39.5% patients and 49.7% patients recurred in the CRT group and CT group, respectively (P = 0.011). From INT0116, we found that 42.7% patients recurred in the CRT group which was similar to our study (18). As a local treatment, radiotherapy has advantages for local control. Similarly, several studies have also found that adjuvant CRT significantly reduced the regional failure compared with adjuvant CT (19, 20). In our study, we found that the metastatic recurrence rate was higher in the CRT group than in the CT group; we consider that the possible reason is that 126 recurring patients in the CT group were excluded due to the loss of specific recurrence information when following up by telephone.

A previous study has proven that the molecular subtypes varied in the different locations of gastric cancer (21). Zhao et al. (22) analyzed 6,479 cases of proximal gastric cancer patients and 9,640 cases of distal gastric cancer patients retrospectively. They found more advanced T and N stages in the proximal location compared with distal location. However, there is no significant survival prognosis between two groups. Similarly, in our study, although the location of the tumor was different in the CRT and CT group, there was no significant relationship between the tumor location and overall survival in the univariate Cox regression analysis (P = 0.454). Our study has three limitations. Firstly, during the follow-up, 8.2% recurrent patients in the CRT group and 42.0% recurrent patients in the CT group underwent imaging or the pathological test in other hospitals which resulted into the missing of recurrence patterns. Secondly, tumor location and age cannot be balanced simultaneously during the PSM. Thirdly, the chemotherapy regime varied and the adverse events or nutritional status was not evaluated in this study.

In conclusion, for stage III gastric cancer or gastroesophageal junction cancer after D2/R0 resection, adjuvant chemoradiotherapy could improve the overall survival and regional control compared with adjuvant chemotherapy. Further studies should be designed to explore the benefit of radiotherapy in the preoperative treatment for locally advanced gastric cancer.

## Data Availability Statement

The raw data supporting the conclusions of this article will be made available by the authors, without undue reservation.

## Ethics Statement

Ethical review and approval were not required for the study on human participants in accordance with the local legislation and institutional requirements. Written informed consent for participation was not required for this study in accordance with the national legislation and the institutional requirements.

## Author Contributions

JMS and WZK: data collection, investigation, data analysis, and writing of the manuscript. YT and NL: great effort in the radiotherapy for patients and review of the manuscript. LMJ: judgement of the images and review of the manuscript. LY: great effort in the chemotherapy for patients and review of the manuscript. SLW, YWS, YPL, HF, NNL, SNQ, BC, and YXL: designing and reviewing of the manuscript. YTT: data collection, formal analysis, project administration, patient care, and reviewing of the manuscript. JJ: data collection, formal analysis, statistical analysis guidance, project administration, patient care, and editing of the draft of the manuscript. All authors read and approved the final manuscript.

## Funding

This study is supported by the Beijing Hope Run Special Fund of Cancer Foundation of China (No. LC2018L03) and National Natural Science Foundation of China (82073352).

## Conflict of Interest

The authors declare that the research was conducted in the absence of any commercial or financial relationships that could be construed as a potential conflict of interest.

## Publisher’s Note

All claims expressed in this article are solely those of the authors and do not necessarily represent those of their affiliated organizations, or those of the publisher, the editors and the reviewers. Any product that may be evaluated in this article, or claim that may be made by its manufacturer, is not guaranteed or endorsed by the publisher.
